# Phosphogypsum and Borogypsum as Additives for Sustainable and High-Performance 3D-Printable Concrete

**DOI:** 10.3390/polym17182530

**Published:** 2025-09-19

**Authors:** Yeşim Tarhan, Berrin Atalay

**Affiliations:** 1 Technical Sciences Vocational School, Ardahan University, 75002 Ardahan, Türkiye; 2 Graduate Institute of Advanced Technologies, Ardahan University, 75002 Ardahan, Türkiye

**Keywords:** 3D-printed concrete, phosphogypsum, borogypsum, fly ash, GGBS, polypropylene fiber, sustainability

## Abstract

3D-printable concretes often require high binder content. This study evaluates the use of industrial gypsum by-products, phosphogypsum (PG) and borogypsum (BG), as partial cement replacements to enhance sustainability without compromising printability. PG and BG were incorporated at 2.5–10 wt% to replace the gypsum fraction in cement-based mortars containing fly ash (FA) or ground granulated blast-furnace slag (GGBS), with and without fibers. The fresh properties (spread flow diameter, open time, air content, density, and pH) and compressive strength were measured. At 28 days, the highest strength was achieved with a 7.5% PG addition to the GGBS system (~51 MPa), which exceeded the strength of the GGBS control C1 (~47.6 MPa). In the FA system, 2.5% PG reached 42.5 MPa, comparable to the FA control C2 (41.2 MPa). BG caused pronounced strength penalties at ≥7.5% across both binder systems, indicating a practical BG ceiling of ≤5%. Open time increased from ~0.75 h in the controls to ~2–2.5 h in BG-FA mixes with fibers, whereas PG mixes generally maintained a stable, printable window close to control levels. Overall, adding 5–7.5% PG, particularly in the presence of GGBS, improved mechanical performance without compromising workability. However, BG should be limited to ≤5% unless extended open time is the primary objective. These findings provide quantitative guidance on selecting PG/BG dosages and FA/GGBS systems to balance strength and printability in cement-based, 3D-printable concretes.

## 1. Introduction

Additive manufacturing, otherwise known as 3D printing, is transforming the construction industry by replacing labour-intensive formwork with automated layer-by-layer deposition, trimming material waste to the extruded layer, shortening project timelines through on-site fabrication [1,2,3,4,5,6], and unlocking geometries that conventional casting cannot achieve. These geometries may include biomimetic shells and bespoke infill patterns [7,8,9]. However, these advantages are dependent on a delicate rheological balancing act: the fresh cementitious mixture must be fluid enough to flow smoothly through pumps, hoses, and nozzles under relatively low pressures; cohesive enough to maintain a continuous filament without segregation or bleeding; and sufficiently thixotropic to gain yield stress within seconds so that each new layer can bear the increasing dead load of the structure while resisting deformation from subsequent passes [10,11,12]. The simultaneous necessity for pumpability, extrudability, buildability, and shape stability of 3D-printable concrete (3DPC) constitutes the so-called “rheological paradox”, which represents a significant impediment to the advancement of this technology from laboratory prototypes to full-scale structural applications [13].

To reconcile the opposing requirements of flowability (for continuous pumping and smooth nozzle discharge) and buildability (for rapid gain of green strength that resists self-weight deformation), mix designers increasingly exploit supplementary cementitious materials (SCMs). Fly ash (FA), silica fume (SF), and ground granulated blast-furnace slag (GGBS) not only displace energy-intensive clinker but also fine-tune rheology. The hollow, glassy cenospheres of fly ash act as micro-ball bearings that lower plastic viscosity and improve pumpability [14,15,16,17,18]. The ultrafine amorphous silica in silica fume sharply increases yield stress and thixotropic recovery, enhancing interlayer adhesion; however, excessive SF (>10–12 wt%) can impede extrusion [19,20,21]. GGBS’s latent-hydraulic glass accelerates early strength gain but shortens open time, potentially limiting large-scale print windows [22,23]. Metakaolin, which is characterised by its high content of reactive alumina, has been demonstrated to enhance buildability and long-term strength [24,25]. However, it has also been observed to increase water demand, necessitating precise dosing of admixture [26]. Designers often blend two or more SCMs to create a synergistic rheology profile that satisfies extrusion criteria while cutting up to 50% of the cement content [27]. Fine adjustments are then made with viscosity-modifying or high-range water-reducing agents; however, these admixtures add cost, introduce extra embodied CO_2_, and—particularly for polycarboxylate superplasticizers—can delay the early stiffening required for tall builds [19]. As a result, there is growing interest in “admixture-lean” formulations that achieve the same rheological targets through particle-packing optimisation and targeted gypsum additions, thereby advancing both the technical and environmental sustainability of 3D-printable concrete.

Natural gypsum (CaSO_4_·2H_2_O) calcines at just ~150 °C, offering a low-carbon, fast-hardening binder that has long been added to Portland cement to moderate C_3_A hydration; however, its function as a principal rheology modifier in extrusion-based 3DPC is only beginning to be mapped. Peng et al. [28] declared fewer than a dozen studies on gypsum-rich mixtures for additive manufacturing, most limited to plaster-type pastes rather than structural mortars. Among the few structural attempts, Sahmenko et al. [29] demonstrated that ternary gypsum-cement-pozzolan blends can reach 37 MPa while retaining the rapid set needed for layer build-up, but they did not quantify the resulting yield-stress–viscosity profile that governs filament stability.

PG and BG, by-products of the manufacture of phosphoric acid and boric acid, respectively, represent a substantial under-utilised gypsum resource. Both consist predominantly of calcium sulfate hemihydrate (CaSO_4_·½H_2_O), but they also contain impurities derived from the manufacturing process, such as fluoride (F^−^), phosphate (PO_4_^3−^), and boron-bearing species (e.g., B_2_O_3_). Depending on the dosage and mixing conditions, these impurities can either retard or accelerate cement hydration, modify setting time, and affect early-age strength and microstructure. PG is produced at a rate of around 5 tonnes for every tonne of phosphoric acid [30], while BG is produced during boric acid production [31].

A growing body of research on eco-efficient binders suggests that, in cast concretes, PG additions up to 10 wt% can function as a latent hydraulic component, effectively participating in secondary cementitious reactions and contributing to strength gain [29]. Recent advancements have demonstrated the potential of PG-based mixtures to achieve 28-day compressive strengths more than 30 MPa, even in the absence of chemical admixtures. This suggests that PG has considerable promise as a partial cement replacement for the next generation of low-clinker, environmentally friendly concretes [32]. The main mechanisms are believed to involve both the formation of ettringite and the stimulation of pozzolanic or latent hydraulic reactions in the presence of supplementary cementitious materials (SCMs) such as fly ash or slag.

Conversely, the extant literature concerning borogypsum remains relatively sparse. Most studies have focused on its use in cast mortars, where BG additions greater than 5 wt% have been found to prolong the setting time and result in only modest strength improvements or, in some cases, slight reductions in mechanical performance [33,34]. The precise influence of BG’s boron content and associated ions on cement hydration and microstructure development is not yet fully understood, especially in complex multi-component systems. Furthermore, to the best of the present authors’ knowledge, no studies have systematically examined the behaviour of borogypsum under the distinctive rheological conditions of 3D printing—namely, high shear rates, thixotropic rebuilding, and rapid strength gain required for successful layer-by-layer deposition.

Despite the documented enhancement of strength and set control in cast concrete exhibited by PG and BG, their utilisation in structural 3DPC remains virtually unexplored. Given this context, there remains a critical need to evaluate the potential of these industrial gypsum by-products in extrusion-based, 3DPC. Understanding how their unique chemical profiles interact with other SCMs and affect the fresh and hardened properties of the mixture is essential for realizing their valorisation in advanced construction technologies.

To the best of our knowledge, this is one of the first studies to systematically evaluate 3D-printable, cement-based concrete incorporating industrial gypsum by-products (PG and BG) as partial replacements for gypsum at 2.5%, 5%, 7.5% and 10% by weight, alongside supplementary cementitious materials (FA or GGBS). A comprehensive assessment was conducted to evaluate the impact of these gypsums on various parameters, including pumpability, flow behaviour, open time, buildability, air content, pH, compressive strength over a period of 1–56 days, and 24-h capillary absorption. Using only a low-dosage polycarboxylate ether (PCE) superplasticizer (1% by weight of binder), up to 40 wt% clinker was replaced by gypsum + SCMs without compromising printable rheology. It has been demonstrated that industrial gypsums function as natural rheology modifiers and latent binders, thereby enhancing both the environmental sustainability and mechanical performance of 3D-printable concrete.

## 2. Experimental Methodology

### 2.1. Materials and Mix Design

In this study, 3D-printable concrete mixtures were produced using CEM II/B-LL 42.5 R white cement, conforming to the specifications of TS EN 197-1 [35]. Granulated blast-furnace slag (GGBS), fly ash (FA), and calcined kaolin clay were incorporated as supplementary cementitious materials (SCMs), with their properties detailed in Table 1. Additionally, Plaster of Paris (CaSO_4_·½H_2_O) was used as a secondary binder to reduce cement content while enhancing shape stability and printability.

The fine aggregate system consisted of a fixed combination of marble dust and quartz sands. Marble dust was used as a filler material, accounting for 5% of the total aggregate volume. Quartz sands with particle size ranges of 0–0.5 mm, 0.4–0.8 mm, and 0.5–1.2 mm were incorporated at proportions of 30%, 30%, and 35%, respectively. The quartz sand had a specific gravity of 2.65 g/cm^3^, while the marble dust had a specific gravity of 2.70 g/cm^3^.

The total binder content of all the mixtures was set to 500 kg/m^3^. Two control groups were established, incorporating either granulated blast-furnace slag (GGBS) or fly ash (FA) as the supplementary cementitious material. The total binder dosage is 500 kg/m^3^, comprising 60% cement (300 kg), 20% SCM (100 kg, consisting of GGBS or FA), and 20% gypsum (100 kg). Calcined kaolin clay has been added at a constant dosage of 5% of the total binder dosage to ensure shape stability and facilitate workability. A water-to-binder ratio of 0.38 was maintained across all formulations to ensure uniformity in the fresh state. To adjust the workability of the mixtures with a high binder content, a polycarboxylate-based superplasticizer (SIKA Viscocrete ACE 450) was used at 1% of the total binder weight.

PG and BG were incorporated as partial gypsum substitutes at replacement levels of 2.5%, 5%, 7.5% and 10% by mass. The influence of fiber reinforcement was also investigated to enhance shape retention and interlayer bonding. Polypropylene fibers (5 mm long) were added at a dosage of 0.2% of the total concrete volume as a variable parameter. All mix proportions were prepared for a batch volume of 1 m^3^. In the naming convention, mixtures incorporating GGBS are denoted directly, while those containing fly ash are identified with the “-FA” suffix. The primary control mix, designated as C1, includes GGBS, whereas the secondary control mix, C2, includes FA.

Mixtures are labelled by replacement level and gypsum type, with ‘-F’ indicating fiber addition and ‘-FA’ indicating fly ash. For example, 2.5BG-F represents a GGBS-based mix with 2.5% borogypsum and fibers; 5PG denotes a GGBS-based mix with 5% phosphogypsum and no fibers; and 2.5BG-FA-F identifies a fly-ash-based mix with 2.5% borogypsum and fibers.

**Figure 1:**
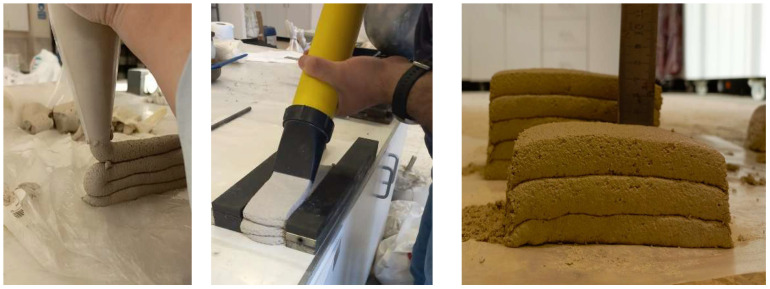
Evaluating the printability of 3D-printable concrete mixtures.

All mixtures were prepared using a 5-L Hobart planetary mixer. First, the dry ingredients (cement, gypsum, SCMs, calcined clay, and aggregates) were mixed for three minutes at low speed (60 rpm) without adding water. Then, half of the mixing water was added, and the mixture was stirred for two minutes at medium speed (80 rpm). The superplasticizer was introduced with the remaining water, and the mixture was then mixed for a further two minutes at high speed (120 rpm). The mixture was then left to rest for one minute to check for any signs of premature setting, which occasionally occurred due to the presence of gypsum. If so, the mixture was remixed at high speed for two more minutes. This second mixing cycle ensured a uniform, workable consistency. Once homogeneous, the fresh mixtures were ready for testing and casting. Printability was evaluated using a mortar injection system, as shown in Figure 1.

All mixtures were prepared by dry-mixing powders and aggregates and adding water and superplasticizer. The fresh mixtures were cast in two layers into standard molds, lightly tamped, and leveled. Specimens were left in molds for 24 h, then demolded and placed in a curing chamber at 23 ± 2 °C and 80 ± 5% relative humidity until the designated testing age. All mechanical and durability tests were conducted on samples prepared and cured using this procedure.

### 2.2. Experiments

Experimental tests were conducted to evaluate the mechanical and durability-related properties of the 3D-printable concrete mixtures in their fresh and hardened state. All tests were performed in accordance with the relevant EN, TS EN, or ASTM standards, as detailed below.

#### 2.2.1. Flow Table Test

**Figure 2:**
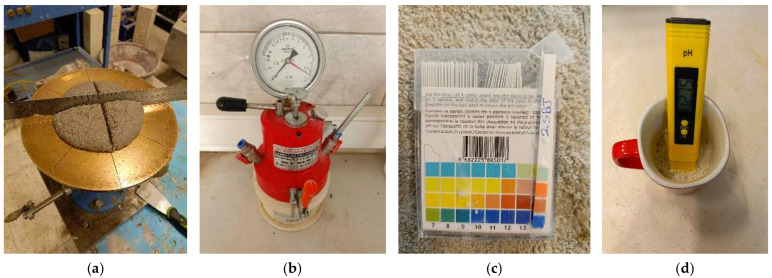
Fresh state testing procedures: (**a**) flow table test used to evaluate flowability and workability; (**b**) air content measurement using a pressure-type apparatus; (**c**) pH measurement with litmus paper; and (**d**) pH measurement using a digital pH meter.

The flowability of the mixtures was evaluated using a flow table test in accordance with TS EN 12350-5 [36], as this is a critical parameter for 3D printability. Before testing, the test apparatus, including the conical mould and flow table surface, was lightly lubricated to minimize friction. The concrete was placed in the mould in two layers, each of which was gently rodded to eliminate air pockets. The mould was then carefully lifted vertically, and the table was dropped 15 times from a prescribed height. The resulting spread diameter was measured along two perpendicular axes using a ruler, as shown in Figure 2a, and the average value was recorded as the flow diameter.

The flow table test was selected to evaluate the workability and extrudability of the 3D-printable mixtures, as it provides a more reliable measure of the deformation and flowability of low-slump, highly thixotropic cementitious materials compared to the conventional slump cone method. This approach is consistent with recent research on 3D-printable mortars and concretes, where flow table spread is considered a key indicator of printability [37].

#### 2.2.2. Open Time Test (Workability)

The open time, defined as the duration during which the mixture retains printable consistency, was evaluated using the flow table test. After the initial measurement, the fresh concrete was stored in a sealed container covered with a damp cloth, and the spreading diameter was re-measured at 15-min intervals following the same procedure. Workability time was determined as the elapsed period until a 3 cm reduction in spread diameter was observed. This threshold was selected based on preliminary extrusion trials, which showed that such a reduction consistently marked the transition from stable to unstable filament formation. At this point, irregularities such as discontinuous deposition and roughness were observed, indicating the practical limit of workable consistency for 3D printing. This approach aligns with previous studies that employed flow diameter reduction as a means of assessing workability retention in cementitious systems [38,39,40].

In the context of extrusion-based 3D printing, open time represents the critical window in which the mixture must simultaneously satisfy two conditions: sufficient fluidity to permit continuous pumping and extrusion, and adequate thixotropic build-up to ensure that deposited layers can support subsequent layers without deformation. If the open time is too short, premature stiffening disrupts extrusion and causes nozzle blockages. Conversely, if it is too long, structural build-up is delayed, resulting in deformation or collapse of printed layers under their own weight. The 3 cm reduction criterion thus provides a reproducible and experimentally validated indicator of this balance, defining the workable window in which 3D-printable mortars can achieve both printability and buildability.

#### 2.2.3. Air Content

The air content of the fresh concrete was determined using a pressure-type air content meter, conforming to TS EN 12350-7 [41] as shown in Figure 2b. The concrete was slowly placed in the mould. The mould was 12 cm wide and 8.9 cm tall. Each layer of concrete was made firm by tapping it lightly. Once the concrete surface was level with the mould, the top cover of the device was closed, and water was added through the pipes on the sides. Once the valves on the pipes had been closed, air was pumped into the device, and this process continued until the manometer needle reached the specified value. The air pumping process was stopped, and the air was discharged using the lever at the top of the device. The value indicated by the needle was then read and recorded as the air content.

#### 2.2.4. Unit Volume Weight (Fresh Density)

Unit volume weight (or fresh density) was measured concurrently with the air content test, in accordance with TS EN 12350-6 [42]. The container was first tared, then filled in layers with fresh concrete, each layer compacted uniformly. After leveling the top surface, the total weight was measured. Fresh density was calculated by dividing the net weight of the concrete by the known volume of the container.

#### 2.2.5. pH Test

In order to assess the potential influence of phosphogypsum and borogypsum on the chemical environment of the mixtures, pH was measured using two independent methods: a digital pH meter and litmus paper. Concrete samples were prepared by mixing with distilled water to form a slurry, and pH readings were recorded using both techniques to verify accuracy and consistency, as shown in Figure 2c,d.

High alkalinity (pH > 12.5) is necessary to stabilize calcium hydroxide and to promote the dissolution of supplementary cementitious materials such as fly ash and slag, thereby supporting both pozzolanic and latent hydraulic reactions. Maintaining a pH near 13 is also critical for the continuous nucleation and growth of calcium silicate hydrate (C–S–H), which directly controls strength development. Since PG and BG exhibit acidic characteristics, observing the pH of the mixtures therefore provides valuable insight into the alkaline environment, the dissolution of clinker phases and SCMs, and the interpretation of subsequent hydration and strength evolution.

#### 2.2.6. Compressive Strength

**Figure 3:**
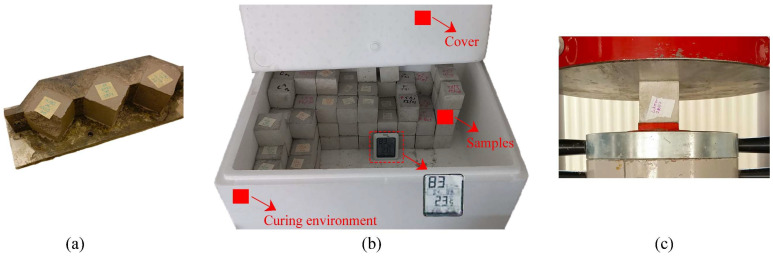
Compressive strength testing process: (**a**) demolding of concrete specimens; (**b**) view of produced cube samples, curing environment; and (**c**) compressive strength testing using a calibrated testing machine.

Compressive strength tests were conducted in accordance with TS EN 12390-3 [43] to evaluate the mechanical performance of the mixtures. Cube specimens measuring 50 × 50 × 50 mm were prepared from each mixture. Compressive strength was measured using a calibrated compression testing machine (Figure 3) at 1, 3, 7, 14, 28 and 56 days. For each age, the mean of three 50 mm cubes (n = 3) was reported.

In this study, compressive strength was selected as the primary experimental parameter because it offers a reliable measure of hydration progress, microstructural evolution, and structural capacity in 3D-printable cementitious mixtures. While other mechanical and durability properties are equally important, the scope of the present work was limited to compressive strength due to the extensive number of formulations that were tested. The development of strength over both early age and long-term periods (1–56 days) was subject to rigorous monitoring, thereby facilitating consistent and comparative evaluation of the PG and BG additions in GGBS- and FA-based systems.

#### 2.2.7. Capillary Water Absorption Resistance

**Figure 4:**
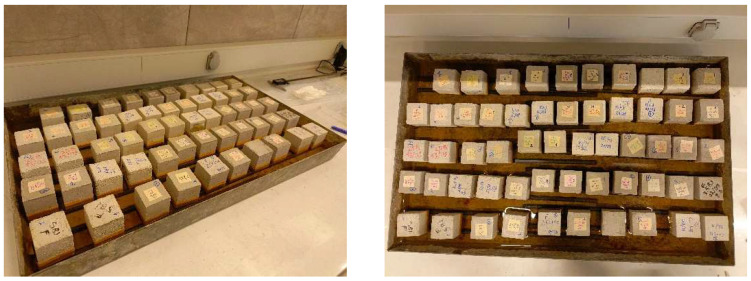
Capillary water absorption resistance test procedure.

To evaluate the capillary absorption behaviour of the mixtures, two specimens from each group were selected and dried in an oven for 24 h. Afterwards, the lateral faces of the specimens were sealed with impermeable tape, leaving only the bottom surface exposed to permit unidirectional water ingress. The initial dry weights of the samples were recorded prior to testing. The specimens were then placed in a capillary water absorption container, ensuring that their bottom surfaces were in contact with water. Mass measurements were taken at the following predetermined time intervals: 12, 30, 60, 120 and 240 min, as well as after 24 h. This allowed the tracking of water uptake over time. The test procedure is illustrated in Figure 4.

## 3. Results and Discussion

### 3.1. Assessment of Fresh Properties Results

Table 2 summarises the fresh-state properties of BG and PG mixtures. Open time (workability) is the time to a 3 cm decrease from the initial spread.

#### 3.1.1. Fresh Density Results

**Figure 5:**
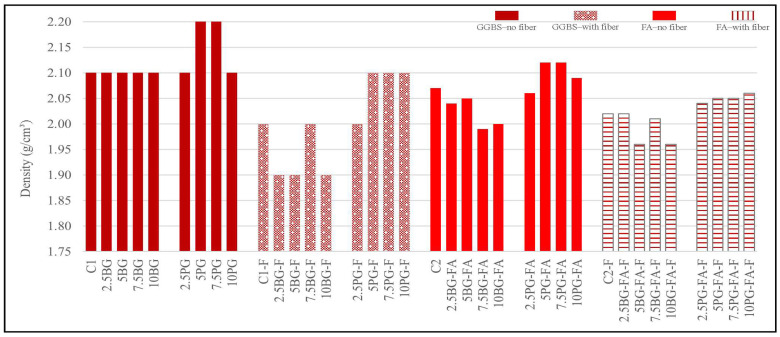
Fresh density results.

As shown in Figure 5, the fresh density of all the mixtures was within the typical range for normal concrete, ranging between 1.9 and 2.2 g/cm^3^. The addition of polypropylene fibers reduced the fresh density slightly across all groups. The highest densities were observed in the GGBS-based mixtures incorporating phosphogypsum, particularly in the 5PG and 7.5PG groups, which reached 2.2 g/cm^3^. These were followed by the fly ash-based phosphogypsum mixtures, with the 5PG-FA and 7.5PG-FA groups exhibiting fresh densities of approximately 2.12 g/cm^3^. In contrast, the density of the GGBS-based mixtures was not significantly affected by the incorporation of borogypsum. Overall, the addition of phosphogypsum tended to increase the fresh density in both GGBS- and FA-containing mixtures.

#### 3.1.2. Air Content Results

**Figure 6:**
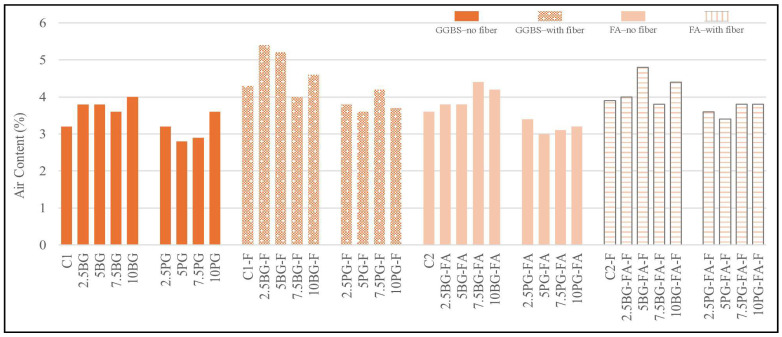
Air content results.

As can be seen in Figure 6, the air content values of the produced mixtures ranged from 2.8% to 5.3%, which falls within the acceptable range for 3D-printable cementitious materials. The control mixtures without fibers (C1 and C2) exhibited baseline values of approximately 3.7% and 3.5%, respectively. These results are consistent with the typical air content range reported in the literature, where values between 2% and 4% are essential for achieving adequate pumpability and extrudability without compromising mechanical performance or interlayer bonding [44,45,46]. The inclusion of polypropylene fibers was found to consistently increase the air content across all mixtures, with the highest value observed in the C1-F group, which exceeded 5%. This increase is attributed to fiber networks promoting air entrapment during mixing, as noted by [44].

Among the borogypsum-modified mixtures, the GGBS-based groups containing fibers—particularly the 5BG-F and 7.5BG-F samples—exhibited the highest air contents, likely due to the combined effects of fiber-induced turbulence and the influence of borogypsum on the matrix structure. In contrast, phosphogypsum-modified mixtures showed stable air content values regardless of the level of replacement. FA-based mixtures demonstrated relatively consistent air content and were less sensitive to gypsum type and fiber inclusion. Overall, borogypsum led to greater variability in air content than phosphogypsum, while the addition of fibers universally increased the amount of entrapped air.

#### 3.1.3. Flow Table Test Results

**Figure 7:**
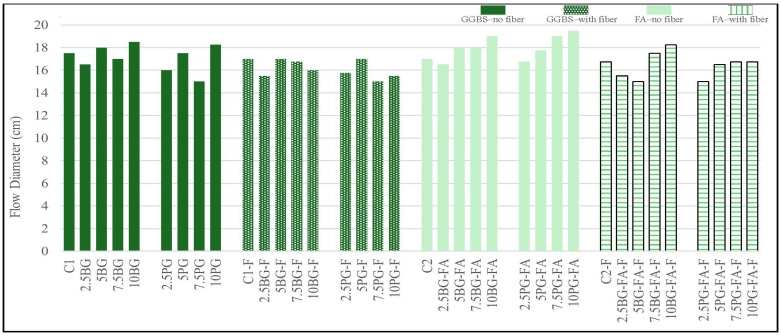
Spread diameter (from flow table test) results.

As shown in Figure 7, the spread diameters of all tested mixtures ranged from 15.0 cm to 19.5 cm. According to [47], slump flow values between 130 and 210 mm are considered suitable for 3D printing applications, while [37] identified a printability range of 18 to 24 cm using flow table tests. Therefore, all mixtures in this study demonstrated acceptable flowability for extrusion-based 3D concrete printing. Among them, the 10PG-FA mixture showed the highest flowability at 19.5 cm, followed by the 7.5PG-FA and 10BG-FA mixtures at 19.0 cm. This trend indicates that increasing the phosphogypsum content, especially in FA-based systems, enhances the deformability of the mixtures, potentially improving their pumpability and extrudability.

In contrast, the lowest spreading diameter values (15.0 cm) were observed in the 5BG-FA-F and 7.5PG mixtures, indicating a more rigid consistency. It is notable that mixtures incorporating polypropylene fibers tended to show reduced spreading diameters, as fibers can form internal networks that resist deformation and hinder flow.

BG-based mixtures showed inconsistent flowability depending on dosage and fiber presence. However, PG-modified mixtures generally enhanced the spread diameter, particularly at moderate to high contents (e.g., 5PG, 10PG and 7.5PG-FA), possibly due to improved paste fluidity. The control mixtures (C1 and C2) without fiber exhibited moderate flow diameters (approximately 17.0–17.5 cm), providing a useful benchmark. The observed range (15–19.5 cm) aligns well with the typical window required for printable cement-based composites, ensuring a balance between pumpability and buildability

#### 3.1.4. Open Time (Workability) Results

In a study by Kim et al. [38] the slump loss of cement mortars incorporating polynaphthalene sulfonate-based superplasticizers was monitored over 90 min using a mini-cone apparatus. Similarly, Alhozaimy et al. [39] investigated the slump retention of concrete containing limestone powder by measuring the initial slump with the standard cone apparatus [40], followed by measurements at 15, 30, 60, 90, 120, and 150 min. These studies contributed to defining the concept of “open time,” referring to the duration in which fresh concrete maintains acceptable workability. Accordingly, assessing the change in spreading diameter over time using the flow table test is a widely adopted method for evaluating workability time in literature.

**Figure 8:**
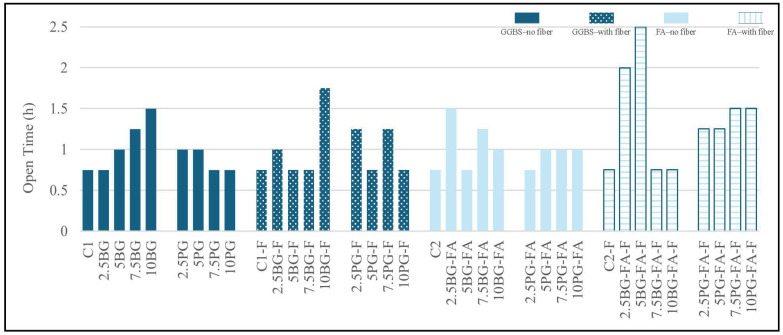
Open time results.

Figure 8 shows the open time of each mixture, which is defined as the duration during which the fresh mix retains its printability without segregating or collapsing. Across all mixtures, workability times ranged from 0.75 to 2.5 h, which is adequate for layer-by-layer deposition within practical 3D printing time frames. The open time was ~0.75 h in the controls (C1 and C2). BG, especially in FA mixes with fibers, extended the window to ~2–2.5 h; PG generally kept timings near control levels. Increases in air content correlate with later strength losses; density trends mirror this behaviour.

Mixtures containing BG and fibers tended to display reduced workability, likely due to faster stiffening induced by a higher surface area and the increased demand for water from fiber networks. Notably, the 10BG-F group-maintained printability for 1.75 h-the longest among borogypsum–fiber combinations—whereas other BG–fiber mixtures remained below one hour.

In contrast, PG mixtures without fiber exhibited relatively uniform workability times of around 0.75 to 1 h. Adding fibers to these groups slightly improved their stability over time, with values of up to 1.5 h. Overall, fly ash-based mixtures displayed more prolonged workability, particularly when combined with phosphogypsum and fibers. These results suggest that an optimal binder composition and controlled fiber dosage can extend the open time of printable mortars, enabling continuous extrusion during printing operations.

Among all tested formulations, the GGBS-based mixture with 5% PG and the FA-based mixture with 2.5% phosphogypsum (2.5PG-FA) demonstrated optimal workability, moderate air content, and consistent flowability, making them strong candidates for practical 3D printing applications.

In 3D printing, shorter open times are advantageous as they indicate faster structural build-up and enhanced dimensional stability, thereby reducing the risk of layer deformation or collapse during printing. Since both PG and BG have set-retarding effects, the most promising mixtures were those that achieved a balance, providing sufficient delay to maintain workable consistency while enabling early stiffening to ensure shape retention.

#### 3.1.5. pH Test Results

**Figure 9:**
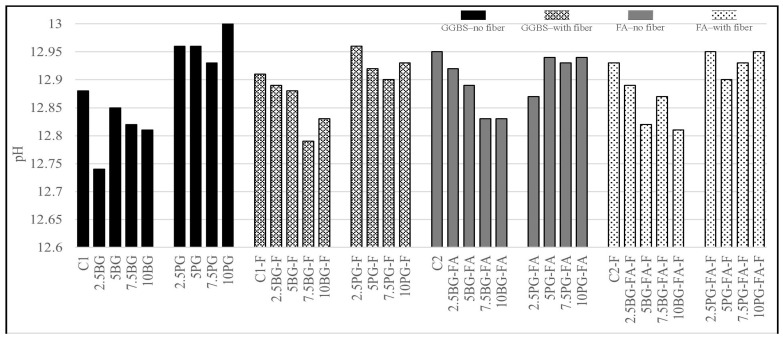
pH meter test results.

As can be seen from Table 2, the pH values of all the mixtures that were tested using litmus paper were found to be 13, which confirms their highly alkaline nature. Figure 9 shows the pH values of the groups measured with the pH testing device.

All mixtures exhibited pH values between 12.7 and 13.0, which is typical of cementitious systems. Despite concerns that gypsum-based additives might reduce alkalinity, the mixtures maintained a pH level within the expected range. Replacing gypsum with phosphogypsum led to a slight increase in pH, indicating its minor contribution to the overall alkalinity. Conversely, the incorporation of borogypsum had a relatively neutral or slightly reducing effect. Notably, the addition of polypropylene fibers appeared to have a slight influence on the pH values across both binder systems, potentially due to minor alterations in the pore solution environment. Overall, all mixtures retained the alkaline conditions essential for proper cement hydration and early-age strength development.

The highly alkaline conditions observed (pH 12.7–13.0) ensured favorable environments for cement hydration and secondary reactions involving SCMs. The slight increase in pH with PG additions is consistent with the accelerated strength development observed in GGBS-based systems, as enhanced alkalinity promotes C–S–H nucleation and ettringite formation. Conversely, the neutral or slightly reducing effect of BG on pH aligns with its known hydration-retarding behavior, which contributed to delayed early-age strength in BG-modified mixtures.

### 3.2. Assessment of Hardened Properties Results

#### 3.2.1. Compressive Strength Test Results

Within the scope of this study, the compressive strength of concrete samples produced from 3D-printable mixtures was evaluated at 1, 3, 7, 14, 28, and 56 days. This comprehensive testing schedule enabled the characterization of both early-age and long-term strength development for mixtures incorporating gypsum, phosphogypsum, and borogypsum. Early-age strength is particularly critical for 3D-printable mixtures, as it directly influences the buildability and structural stability of the printed layers during the additive manufacturing process (Le et al. [48]; Sanjayan et al. [49]). Inadequate early strength may lead to deformation, layer collapse, or instability during or immediately after printing.

Moreover, assessing strength at later ages provided insights into the durability and long-term performance of alternative binder systems that include industrial by-products. The comparison between BG and PG-based formulations revealed distinct strength evolution trends influenced by the binder type, pozzolanic activity, and presence of polypropylene fibers.

**Figure 10:**
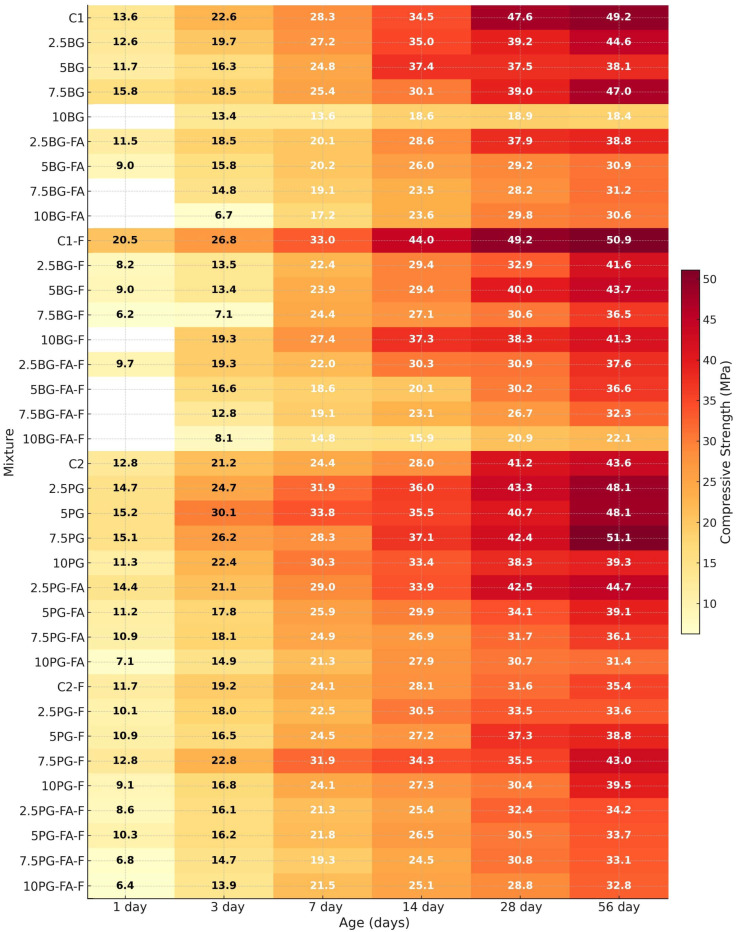
Compressive strength evolution for all mixtures (1–56 days).

For each mix, three 50 mm cubes were tested at every age, and the average value was used to construct the strength-development heat-map in Figure 10. The blank cells (e.g., for 10BG and 7.5BG-FA at 1 day) indicate specimens that could not be demoulded intact because setting had not completed by 24 h; this situation was most frequent in mixes containing higher dosages of borogypsum, fly ash, and fibers, which collectively delayed hydration and early stiffening. The presence of borates significantly retards the hydration of tricalcium silicate and suppresses the formation of calcium hydroxide [50].

As illustrated in Figure 10, the compressive strength of all the tested mixtures was measured at 1, 3, 7, 14, 28, and 56 days. Mixtures incorporating 5–7.5% phosphogypsum achieved early strengths of 14–16 MPa within the first 24 h. Conversely, mixtures comprising 10% borogypsum exhibited considerable setting delays, impeding demolding on the first day, thereby validating the hypothesis that high boron content significantly restricts early hydration [34,51,52].

At intermediate ages (3–14 days), phosphogypsum mixtures demonstrated accelerated strength gain. The GGBS-based formulation with 7.5% phosphogypsum achieved a maximum of ~37 MPa by day 14. This behaviour is indicative of the sulfate-rich nature of phosphogypsum, which has been shown to promote calcium silicate hydrate (C-S-H) nucleation and activate latent hydraulic binders such as slag and fly ash [53,54,55]. Conversely, borogypsum introduces elevated concentrations of boron, thereby impeding hydration through its interference with the reactivity of calcium and aluminate [56]. Consequently, borogypsum mixtures exhibited a dose-dependent response, with 2.5–5% replacement levels attaining compressive strengths analogous to the control (approximately 30 MPa at 14 days). However, increasing the dosage to 10% considerably hindered strength development, yielding values below 20 MPa.

The incorporation of polypropylene fibers resulted in enhanced early strength in mixtures with high reactivity. For instance, the C1-F mixture exhibited a strength of over 20 MPa on day 1, likely attributable to enhanced microcrack control and matrix bridging. However, in borogypsum-rich formulations, the addition of fibers resulted in increased air content and porosity, thereby lowering compressive strength, as similarly reported by [57,58]. In this study, “high reactivity” refers to mixtures that exhibited rapid hydration kinetics, characterized by increased early-age strength development, elevated pH values, and accelerated stiffening observed during fresh and hardening state tests.

In the long term (28–56 days), the highest strength (~51 MPa) was achieved by 7.5% PG–GGBS, which outperformed C1 (47.6 MPa). In FA mixes, 2.5% PG reached a strength of 42.5 MPa, which is comparable to that of C2 (41.2 MPa). BG at or above 7.5% produced significant losses in both binder systems, suggesting a practical upper limit of 5% BG. Borogypsum-containing mixtures with ≤5% substitution demonstrated strengths in the 38–45 MPa range, while 10% BG mixtures exhibited a decrease below 30 MPa. The findings of the present study demonstrate that the incorporation of 5–7.5% phosphogypsum in GGBS- or FA-based matrices is an effective strategy to balance early buildability and long-term strength. Conversely, borogypsum should be restricted to ≤5%, particularly when fibers are present, to minimize strength losses due to increased porosity and hydration retardation.

**Figure 11:**
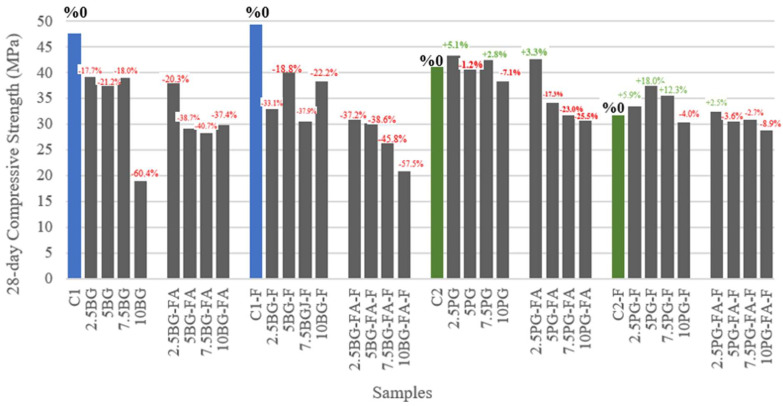
28-day compressive strengths and percent changes compared to controls.

Figure 11 shows the 28-day compressive strengths of all the mixtures that were investigated, alongside their percentage change relative to their respective control groups. This figure allows for a direct comparison of the impact of BG, PG, fiber incorporation, and the type of SCM used (GGBS or FA) on mechanical performance.

The control mixtures (C1 and C2 without fiber, and C1-F and C2-F with fiber) represent the maximum achievable compressive strength under the tested binder systems. C1 (GGBS, no fiber) and C2 (FA, no fiber) achieved 47.6 MPa and 41.2 MPa, respectively, setting robust performance thresholds. These values reflect the typical strength range for printable cementitious composites without waste substitutions, confirming the base matrix’s suitability for 3D concrete printing.

Adding borogypsum at moderate levels (2.5–7.5%) decreased the 28-day compressive strength of both GGBS and FA systems compared to the control, with reductions ranging from approximately 18% to over 40%, depending on the dosage and the presence of fibers. The most severe decline was observed at 10% BG, where strength decreased by over 60% in GGBS and by almost the same amount in FA-based mixes. The strong retarding effect of BG is well documented in the literature. Refs. [34,59] attribute this effect to boron interfering with the reactivity of the calcium and aluminate phases, which inhibits normal hydration and strength gain. Furthermore, adding fiber to BG-rich mixes exacerbated strength losses, likely due to increased entrapped air and weaker fiber-matrix bonding.

By contrast, phosphogypsum additions (2.5–7.5%) tended to maintain or even slightly increase compressive strength relative to the control group, particularly in FA-based systems, both with and without fibers. Notably, the 2.5% PG-FA mixture outperformed the control by 5%, while other PG-FA blends remained within ±5%. GGBS-based mixes with PG substitution exhibited negligible to modest decreases, rarely exceeding 7%. Even at 10% PG, a minor decrease or modest increase was observed, suggesting that the critical threshold for PG addition is higher than for BG. This finding corroborates recent studies [60] which show that moderate levels of phosphogypsum supply sufficient soluble sulfates to promote the formation of calcium silicate hydrate (C-S-H) and ettringite, thereby accelerating hydration and improving both early and long-term strength, particularly in slag-activated systems.

The strength outcomes of fiber inclusion were affected differently depending on the type of gypsum and SCM. In control and PG-based groups, adding polypropylene fibers sometimes had a neutral or slightly positive effect, particularly in FA-rich systems (e.g., 5PG-FA-F, 7.5PG-FA-F), where crack bridging may offset the negative impact of increased air content. Conversely, in BG-rich mixtures, fibers consistently exacerbated strength losses, likely due to poor matrix bonding and increased voids. Notably, the highest positive fiber impact (+18%) was observed in an FA-PG system, suggesting an interaction between fiber, matrix composition, and hydration dynamics.

Mixtures incorporating 5–7.5% phosphogypsum in both GGBS and FA binder systems consistently achieved the highest early and late-age compressive strengths, with 7.5PG and 5PG-FA standing out for their balanced performance. In contrast, mixtures with borogypsum content above 5% are not recommended due to significant strength reductions and delayed setting.

#### 3.2.2. Capillary Water Absorption Resistance Results

Table 3 shows the results for the capillary water absorption resistance of all the mixtures.

**Figure 12:**
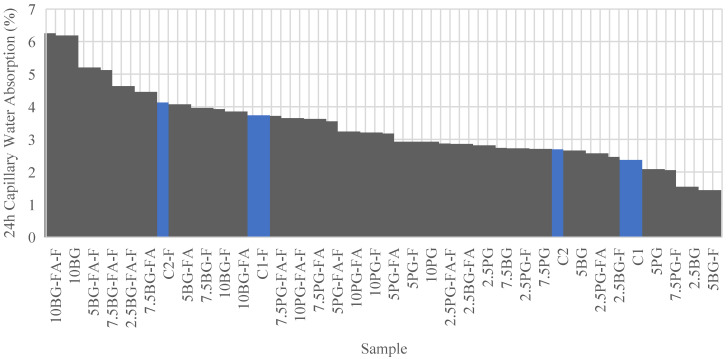
24 h Capillary water absorption resistance (sorted by performance).

Figure 12 illustrates the 24-h percentage weight gain for each sample, clearly showing how resistant they are to water uptake. The control groups are highlighted in blue, while all other mixtures are depicted in grey, making it easy to identify performance differences among the tested formulations.

As shown in Figure 12, the 24-h capillary water absorption rate for the tested mixtures ranged from approximately 1.4 to 6.3 per cent, which is within the typical range observed for conventional concrete. Capillary absorption values for well-compacted cementitious materials are generally found to be within this range; porosity and the content of mineral admixtures significantly influence absorption behaviour [61,62,63].

The lowest water absorption was observed in the 5BG-F mixture (1.44%), followed by the 2.5BG mixture (1.55%). This indicates that certain combinations of borogypsum and fiber can increase the resistance to capillary absorption. In contrast, the highest absorption was recorded in the 10BG and 10BG-FA-F samples (6.19% and 6.25%, respectively), indicating that increased borogypsum content can significantly reduce water resistance, especially when combined with fly ash or additional fibers.

Most of the control and phosphogypsum-modified groups exhibited medium sorption values (between 2.5 and 3.5%), while some PG and GGBS-included mixtures (e.g., 2.5PG, 5PG, and 7.5PG) showed comparable or slightly better water resistance than the controls. However, samples containing higher BG dosages (especially with fiber addition) tended to exhibit high water uptake, potentially limiting matrix densification due to increased porosity and the retarding effect of boron on cement hydration.

Although the addition of polypropylene fibers was beneficial in terms of crack control and mechanical performance, it resulted in increased capillary absorption in most mixes. This observation is consistent with previous studies reporting that fiber networks can open additional pathways [64,65].

The lowest 24-h water absorption was observed in the 5BG-F and 2.5BG mixtures. However, for the best overall balance between strength and durability, the phosphogypsum-based mixtures (notably 5PG and 7.5PG) are preferable, as they combine good capillary resistance with superior compressive strength.

## 4. Conclusions

This study examined the impact of using phosphogypsum and borogypsum as sustainable additives in 3D-printable, cement-based mixtures to improve mechanical performance and reduce environmental impact. The experimental programme evaluated the influence of these industrial by-products on the fresh state properties of the mixtures (including flowability, workability time, air content, pH, and density), as well as their compressive strength development and capillary water absorption resistance. This was achieved using both GGBS- and FA-based binder systems, with and without polypropylene fibers.

The results showed that all the produced mixtures exhibited suitable fresh state properties for 3D printing, with flow diameters, workability times, and air contents that met the established requirements for extrusion-based cementitious materials. Phosphogypsum was particularly effective in maintaining or improving workability and flowability, especially in fly ash-containing systems. Conversely, borogypsum decreased workability at higher dosages due to its hydration-retarding effect.

Compressive strength tests showed that moderate additions of PG (5–7.5%) provided the best balance between early buildability and long-term strength, particularly when combined with GGBS or FA. In contrast, additions of BG above 5% led to a marked reduction in both early-age and 28-day strengths due to delayed hydration, with the most pronounced strength losses occurring at the highest dosages. The incorporation of polypropylene fibers increased early strength and provided additional crack resistance in highly reactive matrices but contributed to increased air content and porosity in BG-rich mixtures, sometimes offsetting the mechanical benefits. This study primarily focused on evaluating the fresh-state properties and compressive strength of 3D-printable mixtures, with tests performed on conventionally cast specimens. Other critical mechanical characteristics, such as flexural strength, fracture resistance, and crack propagation, will be systematically investigated in future research using 3D-printed specimens to provide a more comprehensive understanding of the structural performance of PG- and BG-modified mixtures.

Results from tests on capillary water absorption resistance confirmed that most mixtures achieved 24-h absorption values within the typical range for conventional concretes (1.4–6.3%). Some PG and low-BG mixtures, particularly those containing GGBS, exhibited slightly enhanced water resistance compared to the control group. Conversely, the high-BG and fiber-rich groups displayed increased absorption, potentially due to the combined effects of porosity and delayed matrix densification.

Overall, the findings suggest that phosphogypsum can effectively enhance the sustainability and performance of 3D-printable cementitious composites at medium (2.5–7.5%) dosages, particularly when combined with GGBS or FA. While borogypsum is viable at low dosages (2.5–5%), it should be carefully limited to avoid detrimental effects on strength and durability. These results contribute to the ongoing development of environmentally friendly, high-performance digital construction materials, supporting the recycling of industrial by-products and broader sustainable building practices.

The incorporation of PG and BG as partial cement replacements demonstrates potential for reducing clinker consumption and enhancing resource efficiency in 3D-printable concretes. Although the results indicate a promising direction for sustainable construction materials, a comprehensive environmental assessment that includes CO_2_ emissions, energy demand, and life cycle analysis is still required and will be addressed in future studies.

## Figures and Tables

**Table 1:** Chemical and physical properties of cement, GGBS, FA, and Kaolin Clay.

		Cement	GGBS	FA	Kaolin Clay	Gypsum (Paris Plaster)
*Chemical compositions (%)*	SiO_2_	23–26	36.1	56.8	57.6	2.5
	Al_2_O_3_	3–5	9.8	25.4	26.2	0.39–0.50
	Fe_2_O_3_	≤0.35	1.1	5.8	3.7	0.05–0.1
	CaO	62–64	40.2	3.7	0.3	32.4
	MgO	0.5–1.5	10.3	1.8	0.7	0.9
	SO_3_	3.20–3.80	0.2	0.4	0.07	45.7
	Na_2_O	≤0.06	0.4	0.4	0.4	0.06–0.1
	K_2_O	0.5–0.7	0.8	0.7	3.5	0.05–0.1
	Cl^-^	0.02	-	-	-	-
*Physical properties*	Specific gravity (g/cm^3^)	3.11	2.88	2.24	2.63	2.33
	Blaine specific surface (cm^2^/g)	4000–4500	3300	2800	2110	
	Initial setting time (min)	120–150				
	Final setting time (min)	160–200				
	Compressive strength (MPa)	51–53				

**Table 2:** Fresh State Properties of 3D-Printable Concrete Mixtures.

Mixture	pH Results		Spread (Flow) Diameter (cm)	Workability (Open Time) (h)	Air Content (%)	Fresh Density (g/cm^3^)
	Litmus Paper	pH Meter				
C1	13	12.88	17.5	0.75	3.2	2.1
2.5BG	13	12.74	16.5	0.75	3.8	2.1
5BG	13	12.85	18	1	3.8	2.1
7.5BG	13	12.82	17	1.25	3.6	2.1
10BG	13	12.81	18.5	1.5	4	2.1
C1-F	13	12.91	17	0.75	4.3	2
2.5BG-F	13	12.89	15.5	1	5.4	1.9
5BG-F	13	12.88	17	0.75	5.2	1.9
7.5BG-F	13	12.79	16.75	0.75	4	2
10BG-F	13	12.83	16	1.75	4.6	1.9
2.5PG	13	12.96	16	1	3.2	2.1
5PG	13	12.96	17.5	1	2.8	2.2
7.5PG	13	12.93	15	0.75	2.9	2.2
10PG	13	13	18.25	0.75	3.6	2.1
2.5PG-F	13	12.96	15.75	1.25	3.8	2
5PG-F	13	12.92	17	0.75	3.6	2.1
7.5PG-F	13	12.9	15	1.25	4.2	2.1
10PG-F	13	12.93	15.5	0.75	3.7	2.1
C2	13	12.95	17	0.75	3.6	2.07
2.5BG-FA	13	12.92	16.5	1.5	3.8	2.04
5BG-FA	13	12.89	18	0.75	3.8	2.05
7.5BG-FA	13	12.83	18	1.25	4.4	1.99
10BG-FA	13	12.83	19	1	4.2	2
C2-F	13	12.93	16.75	0.75	3.9	2.02
2.5BG-FA-F	13	12.89	15.5	2	4	2.02
5BG-FA-F	13	12.82	15	2.5	4.8	1.96
7.5BG-FA-F	13	12.87	17.5	0.75	3.8	2.01
10BG-FA-F	13	12.81	18.25	0.75	4.4	1.96
2.5PG-FA	13	12.87	16.75	0.75	3.4	2.06
5PG-FA	13	12.94	17.75	1	3	2.12
7.5PG-FA	13	12.93	19	1	3.1	2.12
10PG-FA	13	12.94	19.5	1	3.2	2.09
2.5PG-FA-F	13	12.95	15	1.25	3.6	2.04
5PG-FA-F	13	12.9	16.5	1.25	3.4	2.05
7.5PG-FA-F	13	12.93	16.75	1.5	3.8	2.05
10PG-FA-F	13	12.95	16.75	1.5	3.8	2.06

**Table 3:** Capillary Water Absorption Resistance Test Results.

Sample	Beginning Weight (g)	Mass at 12 min (g)	Mass at 30 min (g)	Mass at 60 min (g)	Mass at 120 min (g)	Mass at 240 min (g)	Mass at 24 h (g)	Percentage Changes (%)
C1	266.90	268.18	268.78	269.27	269.77	270.55	273.22	2.37
C1-F	269.58	271.15	272.18	272.90	273.76	275.03	279.65	3.74
2.5BG	257.17	258.00	258.36	258.60	259.12	259.57	261.15	1.55
2.5BG-F	261.75	262.99	263.62	263.80	264.48	265.28	268.20	2.46
5BG	267.37	268.66	269.23	269.81	270.53	271.46	274.48	2.66
5BG-F	263.28	264.03	264.40	264.54	264.91	265.34	267.07	1.44
7.5BG	263.05	264.37	265.08	265.56	266.46	267.38	270.27	2.74
7.5BG-F	249.39	251.23	252.22	252.92	253.95	255.18	259.29	3.97
10BG	237.40	240.80	242.66	244.07	245.98	248.11	252.10	6.19
10BG-F	272.79	274.90	276.06	277.08	278.34	279.66	283.52	3.93
2.5PG	273.64	275.37	276.08	276.54	277.41	278.35	281.36	2.82
2.5PG-F	250.19	251.44	252.05	252.53	253.06	253.84	257.01	2.73
5PG	264.65	265.95	266.44	266.91	267.48	268.16	270.18	2.09
5PG-F	241.32	242.35	242.94	243.43	243.98	244.80	248.38	2.93
7.5PG	272.50	273.99	274.83	275.36	276.20	277.14	279.89	2.71
7.5PG-F	255.65	256.65	257.09	257.49	258.18	258.83	260.92	2.06
10PG	263.48	264.65	265.35	265.86	266.72	267.68	271.19	2.93
10PG-F	261.37	262.95	263.62	264.31	265.25	266.33	269.77	3.21
C2	257.50	258.68	259.27	259.78	260.34	261.21	264.44	2.70
C2-F	260.40	262.04	263.29	264.22	265.32	266.69	271.15	4.13
2.5BG-FA	260.81	261.97	262.56	262.97	263.73	264.68	268.26	2.86
2.5BG-FA-F	253.73	251.51	256.43	257.29	258.60	260.21	265.50	4.64
5BG-FA	248.79	250.06	250.89	251.42	252.49	253.76	258.95	4.08
5BG-FA-F	248.30	250.05	251.45	252.40	253.93	255.67	261.23	5.21
7.5BG-FA	253.53	255.27	256.55	257.46	258.82	260.25	264.83	4.46
7.5BG-FA-F	244.45	246.27	247.43	248.34	249.72	251.40	257.00	5.13
10BG-FA	245.52	247.28	248.15	248.80	249.91	251.16	254.98	3.85
10BG-FA-F	242.34	244.76	246.34	247.68	249.42	251.52	257.48	6.25
2.5PG-FA	259.37	260.42	261.21	261.58	262.38	263.24	266.03	2.57
2.5PG-FA-F	247.68	248.76	249.51	249.97	250.78	251.75	254.79	2.87
5PG-FA	251.22	252.22	252.96	253.44	254.29	255.30	259.20	3.18
5PG-FA-F	246.21	247.47	248.14	248.73	249.39	250.43	254.95	3.55
7.5PG-FA	249.82	251.28	252.10	252.76	253.73	254.84	258.89	3.63
7.5PG-FA-F	245.03	246.42	247.13	247.76	248.49	249.54	254.14	3.72
10PG-FA	248.13	249.50	250.07	250.58	251.13	251.96	256.16	3.24
10PG-FA-F	247.10	248.28	248.94	249.54	250.23	251.25	256.13	3.65

## Data Availability

The original contributions presented in this study are included in the article. Further inquiries can be directed to the corresponding author(s).

## References

[1] Hossain M.A., Zhumabekova A., Paul S.C., Kim J.R. (2020). A Review of 3D Printing in Construction and Its Impact on the Labor Market. Sustainability.

[2] Pasco J., Lei Z., Aranas C. (2022). Additive Manufacturing in Off-Site Construction: Review and Future Directions. Buildings.

[3] Ghaffar S.H., Corker J., Fan M. (2018). Additive Manufacturing Technology and Its Implementation in Construction as an Eco-Innovative Solution. Autom. Constr..

[4] Tarhan Y., Şahin R. (2021). Fresh and Rheological Performances of Air-Entrained 3D Printable Mortars. Materials.

[5] Perrot A., Jacquet Y., Caron J.F., Mesnil R., Ducoulombier N., De Bono V., Sanjayan J., Ramakrishnan S., Kloft H., Gosslar J. (2024). Snapshot on 3D Printing with Alternative Binders and Materials: Earth, Geopolymers, Gypsum and Low Carbon Concrete. Cem. Concr. Res..

[6] Tarhan Y., Tarhan İ.H., Perrot A. (2025). Improving Bond Performance of 3D-Printable Earth-Based Mortar Reinforced with Jute Fibers. Chall. J. Struct. Mech..

[7] Guo Z., Niu W., Qi G., Chai G.B., Tai Z., Li Y. (2024). Performance of 3D Printing Biomimetic Conch Shell and Pearl Shell Hybrid Design Composites under Quasi-Static Three-Point Bending Load. J. Mech. Behav. Biomed. Mater..

[8] Ramos A., Angel V.G., Siqueiros M., Sahagun T., Gonzalez L., Ballesteros R. (2025). Reviewing Additive Manufacturing Techniques: Material Trends and Weight Optimization Possibilities Through Innovative Printing Patterns. Materials.

[9] Podroužek J., Marcon M., Ninčević K., Wan-Wendner R. (2019). Bio-Inspired 3D Infill Patterns for Additive Manufacturing and Structural Applications. Materials.

[10] Liu Z., Li M., Weng Y., Wong T.N., Tan M.J. (2019). Mixture Design Approach to Optimize the Rheological Properties of the Material Used in 3D Cementitious Material Printing. Constr. Build. Mater..

[11] Biricik Ö., Mardani A. (2022). Parameters Affecting Thixotropic Behavior of Self Compacting Concrete and 3D Printable Concrete; a State-of-the-Art Review. Constr. Build. Mater..

[12] Lesage K., El-Cheikh K., De Schutter G. (2023). Rheology and processing of cementitious materials. Active Rheology Control of Cementitious Materials.

[13] Lafhaj Z., Rabenantoandro A.Z., el Moussaoui S., Dakhli Z., Youssef N. (2019). Experimental Approach for Printability Assessment: Toward a Practical Decision-Making Framework of Printability for Cementitious Materials. Buildings.

[14] Nodehi M., Aguayo F., Nodehi S.E., Gholampour A., Ozbakkaloglu T., Gencel O. (2022). Durability Properties of 3D Printed Concrete (3DPC). Autom. Constr..

[15] Wang L., Xiao W., Wang Q., Jiang H., Ma G. (2022). Freeze-Thaw Resistance of 3D-Printed Composites with Desert Sand. Cem. Concr. Compos..

[16] Teixeira J., Schaefer C.O., Maia L., Rangel B., Neto R., Alves J.L. (2022). Influence of Supplementary Cementitious Materials on Fresh Properties of 3D Printable Materials. Sustainability.

[17] Xu K., Yang J., He H., Wei J., Zhu Y. (2025). Influences of Additives on the Rheological Properties of Cement Composites: A Review of Material Impacts. Materials.

[18] Boddepalli U., Panda B., Ranjani Gandhi I.S. (2023). Rheology and Printability of Portland Cement Based Materials: A Review. J. Sustain. Cem. Based Mater..

[19] Liu W., Liu X., Zhang L., Wan Y., Li H., Jiao X. (2024). Rheology, Mechanics, Microstructure and Durability of Low-Carbon Cementitious Materials Based on Circulating Fluidized Bed Fly Ash: A Comprehensive Review. Constr. Build. Mater..

[20] Paritala S., Singaram K.K., Bathina I., Khan M.A., Jyosyula S.K.R. (2023). Rheology and Pumpability of Mix Suitable for Extrusion-Based Concrete 3D Printing—A Review. Constr. Build. Mater..

[21] Şahin H.G., Mardani A., Beytekin H.E. (2024). Effect of Silica Fume Utilization on Structural Build-Up, Mechanical and Dimensional Stability Performance of Fiber-Reinforced 3D Printable Concrete. Polymers.

[22] Moula S., Ben Fraj A., Wattez T., Bouasker M., Hadj Ali N.B. (2023). Mechanical Properties, Carbon Footprint and Cost of Ultra-High Performance Concrete Containing Ground Granulated Blast Furnace Slag. J. Build. Eng..

[23] Panda B., Unluer C., Tan M.J. (2018). Investigation of the Rheology and Strength of Geopolymer Mixtures for Extrusion-Based 3D Printing. Cem. Concr. Compos..

[24] Mansour A.M., Al Biajawi M.I. (2022). The Effect of the Addition of Metakaolin on the Fresh and Hardened Properties of Blended Cement Products: A Review. Mater. Today Proc..

[25] Ramezanianpour A.A. (2014). Metakaolin. Cement Replacement Materials.

[26] Wang F., Kovler K., Provis J.L., Buchwald A., Cyr M., Patapy C., Kamali-Bernard S., Courard L., Sideris K. (2018). Metakaolin. RILEM State—Art Rep..

[27] Mishra S.K., Snehal K., Das B.B., Chandrasekaran R., Barbhuiya S. (2025). From Printing to Performance: A Review on 3D Concrete Printing Processes, Materials, and Life Cycle Assessment. J. Build. Pathol. Rehabil..

[28] Peng Y., Unluer C. (2023). Development of Alternative Cementitious Binders for 3D Printing Applications: A Critical Review of Progress, Advantages and Challenges. Compos. B Eng..

[29] Sahmenko G., Puzule L., Sapata A., Slosbergs P., Bumanis G., Sinka M., Bajare D. (2024). Gypsum–Cement–Pozzolan Composites for 3D Printing: Properties and Life Cycle Assessment. J. Compos. Sci..

[30] Pliaka M., Gaidajis G. (2022). Potential Uses of Phosphogypsum: A Review. J. Environ. Sci. Health Part A.

[31] Emrullahoglu Abi C.B. (2014). Effect of Borogypsum on Brick Properties. Constr. Build. Mater..

[32] Sinka M., Vaičiukynienė D., Nizevičienė D., Sapata A., Fornés I.V., Vaitkevičius V., Šerelis E. (2024). Utilisation of By-Product Phosphogypsum Through Extrusion-Based 3D Printing. Materials.

[33] Topçu I.B., Boǧa A.R. (2010). Effect of Boron Waste on the Properties of Mortar and Concrete. Waste Manag. Res..

[34] Sevim U.K., Tümen Y. (2013). Strength and Fresh Properties of Borogypsum Concrete. Constr. Build. Mater..

[35] TS EN 197-1: Cement—Part 1: Composition, Specification and Conformity Criteria for Common Cements. https://www.tse.org.tr/.

[36] TS EN 12350-5: Beton—Taze Beton Deneyleri—Bölüm 5: Yayılma Tablası Deneyi (Testing Fresh Concrete—Part 5: Flow Table Test). https://www.tse.org.tr/.

[37] Papachristoforou M., Mitsopoulos V., Stefanidou M. (2018). Evaluation of Workability Parameters in 3D Printing Concrete. Procedia Struct. Integr..

[38] Kim B.G., Jiang S., Jolicoeur C., Aïtcin P.C. (2000). The Adsorption Behavior of PNS Superplasticizer and Its Relation to Fluidity of Cement Paste. Cem. Concr. Res..

[39] Alhozaimy A.M. (2009). Effect of Absorption of Limestone Aggregates on Strength and Slump Loss of Concrete. Cem. Concr. Compos..

[40] (2020). Standard Test Method for Flow of Hydraulic Cement Mortar.

[41] TS EN 12350-7/AC: Beton—Taze Beton Deneyleri—Bölüm 7: Hava Içeriğinin Tayini—Basınç Yöntemleri (Testing Fresh Concrete—Part 7: Air Content—Pressure Methods). https://www.tse.org.tr/.

[42] TS EN 12350-6: Birim Hacim Kütlesi (Testing Fresh Concrete—Part 6: Density). https://www.tse.org.tr/.

[43] TS EN 12390-3: Beton—Sertleşmiş Beton Deneyleri—Bölüm 3: Deney Numunelerinin Basınç Dayanımının Tayini (Testing Hardened Concrete—Part 3: Compressive Strength of Test Specimens). https://www.tse.org.tr/.

[44] Le T.T., Austin S.A., Lim S., Buswell R.A., Gibb A.G.F., Thorpe T. (2012). Mix Design and Fresh Properties for High-Performance Printing Concrete. Mater. Struct..

[45] Perrot A., Rangeard D., Pierre A. (2016). Structural Built-up of Cement-Based Materials Used for 3D-Printing Extrusion Techniques. Mater. Struct..

[46] Amran M., Abdelgader H.S., Onaizi A.M., Fediuk R., Ozbakkaloglu T., Rashid R.S.M., Murali G. (2022). 3D-Printable Alkali-Activated Concretes for Building Applications: A Critical Review. Constr. Build. Mater..

[47] Tay Y.W.D., Qian Y., Tan M.J. (2019). Printability Region for 3D Concrete Printing Using Slump and Slump Flow Test. Compos. B Eng..

[48] Le T.T., Austin S.A., Lim S., Buswell R.A., Law R., Gibb A.G., Thorpe T. (2012). Hardened properties of high-performance printing concrete. Cem. Concr. Res..

[49] Sanjayan J.G., Nematollahi B., Xia M., Marchment T. (2018). Effect of Surface Moisture on Inter-Layer Strength of 3D Printed Concrete. Constr. Build. Mater..

[50] Bothe J.J., Ceramic P.B.-J. (1999). Kinetics of Tricalcium Aluminate Hydration in the Presence of Boric Acid and Calcium Hydroxide. J. Am. Ceram. Soc..

[51] Boncukcuoğlu R., Yılmaz M.T., Kocakerim M.M., Tosunoğlu V. (2002). Utilization of Borogypsum as Set Retarder in Portland Cement Production. Cem. Concr. Res..

[52] Kavas T., Olgun A., Erdogan Y. (2005). Setting and Hardening of Borogypsum–Portland Cement Clinker–Fly Ash Blends. Studies on Effects of Molasses on Properties of Mortar Containing Borogypsum. Cem. Concr. Res..

[53] Tang L., He Z., Xia Y., Fořt J., Xiang H. (2025). Development of Phosphogypsum-Based Full-Solid-Waste Cementitious Materials: Mechanical Properties, Hydration Mechanisms, and Pollutant Stabilization Mechanisms. J. Build. Eng..

[54] Zhou Z., Li H., Liu N., Peng L., Jiang Z. (2024). Development and Property Optimization of a Sustainable Phosphogypsum-Based Cementitious System with Ground-Granulated Blast Furnace Slag and Carbide Slag. Constr. Build. Mater..

[55] Zhao L., Wang C., Na S., Jin Y., Kang W., Zhu J., Zhang W., Bian Y., Shah S.P. (2025). A Study of Blast Furnace Slag on the Mechanical Properties Improvement and Microstructure of Hemihydrate Phosphogypsum Pretreated by Calcium Hydroxide. Case Stud. Constr. Mater..

[56] Bullerjahn F., Zajac M., Skocek J., Ben Haha M. (2019). The Role of Boron during the Early Hydration of Belite Ye’elimite Ferrite Cements. Constr. Build. Mater..

[57] Falliano D., De Domenico D., Ricciardi G., Gugliandolo E. (2019). Compressive and Flexural Strength of Fiber-Reinforced Foamed Concrete: Effect of Fiber Content, Curing Conditions and Dry Density. Constr. Build. Mater..

[58] Kumar A., Walia B.S., Mohan J. (2006). Compressive Strength of Fiber Reinforced Highly Compressible Clay. Constr. Build. Mater..

[59] Singh N.B., Middendorf B. (2020). Geopolymers as an Alternative to Portland Cement: An Overview. Constr. Build. Mater..

[60] Zhang S., Lu X., Wang J., Deng X., Tan H. (2025). Improving the Properties of Phosphogypsum-Based Clinker-Free Cement System by in-Situ Precipitation of Ettringite Seeds: Strength, Hydration, Microstructure and Sustainability. Mater. Today Commun..

[61] Wang Y., Li L., An M., Sun Y., Yu Z., Huang H. (2022). Factors Influencing the Capillary Water Absorption Characteristics of Concrete and Their Relationship to Pore Structure. Appl. Sci..

[62] Golewski G.L. (2023). Assessing of Water Absorption on Concrete Composites Containing Fly Ash up to 30% in Regards to Structures Completely Immersed in Water. Case Stud. Constr. Mater..

[63] He X., Zeng X., Dong R., Yang J. (2023). Analysis of the Effect of Capillary Water Absorption on the Resistivity of Cementitious Materials. Appl. Sci..

[64] Teixidó H., Staal J., Caglar B., Michaud V. (2022). Capillary Effects in Fiber Reinforced Polymer Composite Processing: A Review. Front. Mater..

[65] Gupta S., Kua H.W., Pang S.D. (2018). Combination of Polypropylene Fibre and Superabsorbent Polymer to Improve Physical Properties of Cement Mortar. Mag. Concr. Res..

